# Comparison of reduced field-of-view DWI and conventional DWI techniques for the assessment of lumbar bone marrow infiltration in patients with acute leukemia

**DOI:** 10.3389/fonc.2023.1321080

**Published:** 2024-01-08

**Authors:** Wenjin Bian, Luyao Wang, Jianting Li, Sha Cui, Wenqi Wu, Rong Fan, Jinliang Niu

**Affiliations:** ^1^ Department of Medical Imaging, Shanxi Medical University, Taiyuan, Shanxi, China; ^2^ Department of Radiology, The Second Hospital of Shanxi Medical University, Taiyuan, Shanxi, China

**Keywords:** diffusion-weighted imaging, reduced field-of-view, acute leukemia, image quality, cellularity

## Abstract

**Objectives:**

To compare the imaging quality, apparent diffusion coefficient (ADC), and the value of assessing bone marrow infiltration between reduced field-of-view diffusion-weighted imaging (r-FOV DWI) and conventional DWI in the lumbar spine of acute leukemia (AL).

**Methods:**

Patients with newly diagnosed AL were recruited and underwent both r-FOV DWI and conventional DWI in the lumbar spine. Two radiologists evaluated image quality scores using 5-Likert-type scales qualitatively and measured signal-to-noise ratio (SNR), contrast-to-noise (CNR), signal intensity ratio (SIR), and ADC quantitatively. Patients were divided into hypo- and normocellular group, moderately hypercellular group, and severely hypercellular group according to bone marrow cellularity (BMC) obtained from bone marrow biopsies. The image quality parameters and ADC value between the two sequences were compared. One-way analysis of variance followed by LSD *post hoc* test was used for the comparisons of the ADC values among the three groups. The performance of ADC obtained with r-FOV DWI (ADC_r_) and conventional DWI(ADC_c_) in evaluating BMC and their correlations with BMC and white blood cells (WBC) were analyzed and compared.

**Results:**

71 AL patients (hypo- and normocellular: n=20; moderately hypercellular: n=19; severely hypercellular: n=32) were evaluated. The image quality scores, CNR, SIR, and ADC value of r-FOV DWI were significantly higher than those of conventional DWI (all *p*<0.05), and the SNR of r-FOV DWI was significantly lower (*p*<0.001). ADC_r_ showed statistical differences in all pairwise comparisons among the three groups (all *p*<0.05), while ADC_c_ showed significant difference only between hypo- and normocellular group and severely hypercellular group (*p*=0.014). The performance of ADC_r_ in evaluating BMC (Z=2.380, *p*=0.017) and its correlations with BMC (Z=-2.008, *p* = 0.045) and WBC (Z=-2.022, *p* = 0.043) were significantly higher than those of ADC_c_.

**Conclusion:**

Compared with conventional DWI, r-FOV DWI provides superior image quality of the lumbar spine in AL patients, thus yielding better performance in assessing bone marrow infiltration.

## 1 Introduction

Acute leukemia (AL) is a malignant clonal disease of hematopoietic stem cells. Leukemia cells proliferate and infiltrate rapidly in the bone marrow, resulting in increased cellularity (1). Diffusion−weighted imaging (DWI) is a magnetic resonance imaging (MRI) technique that can sensitively evaluate tissue pathophysiological changes (2). It has been applied to differential diagnosis, response evaluation and prognosis prediction in hematologic malignancies (3–5). Increased bone marrow cellularity in AL would inhibit the Brownian motion of water molecules, which could be quantitatively reflected by the ADC value obtained from DWI (5–7).

Single-shot echo-planar imaging (SS-EPI) technique is most frequently applied for DWI. However, it is susceptible to magnetic field inhomogeneities, local gradients, as well as chemical shift effects because of its long readout time and low sampling bandwidth in the phase-encode direction (8–10). Anatomical features unique to the spine, such as the air cavities in the abdomen, heterogeneous trabecular bone, and respiratory motion, also pose technical challenges leading to distortion, artifacts, signal pile-ups/dropout, and incomplete fat suppression, which may alter the measurement of quantitative parameter (9–11). Some optimized DWI scanning protocols have been developed to address these issues, including navigated fast spin-echo (12), propeller-based imaging (13), line scan imaging (14), steady-state free precession imaging (15), interleaved (or multishot) (16) and parallel EPI imaging (17). Nevertheless, these techniques also have shortcomings, like penalties in acquisition time, decreased robustness against motion artifacts, reduced signal-to-noise ratio (SNR) per unit time, and specialized coils for parallel EPI (9–11).

In recent years, a promising technique, reduced field-of-view (r-FOV) DWI, has been proposed to improve imaging quality, minimize image distortion, and diminish artifacts. r-FOV DWI applies a 2-dimensional spatially selective echo-planar radiofrequency excitation pulse and a 180° refocusing pulse to reduce the FOV in the phase-encoding direction, which results in a faster k-space traversal and a higher bandwidth in that direction, making it less impressionable to field susceptibility and eddy currents (18–20). Moreover, its excitation protocol allows intrinsic fat suppression (20). r-FOV DWI has been used to various organs with hopeful results (21–31). Previous studies reported that r-FOV technology is feasible for the spine DWI and has higher subjective image quality scores than conventional DWI (19, 20). The lumbar spine is the most commonly used site for AL bone marrow studies due to its regular volume (5, 32). We hypothesized that r-FOV DWI could augment evaluation of lumbar bone marrow in AL by improving image quality.

Therefore, the purposes of this study were to compare the imaging quality of r-FOV DWI with conventional DWI in the lumbar spine of AL patients, and to evaluate whether the ADC value obtained with r-FOV DWI has better performance for assessing bone marrow infiltration in AL.

## 2 Materials and methods

### 2.1 Study participants

This prospective study was approved by the review board of Second Hospital of Shanxi Medical University. Written informed consent was provided by all participants. Between August 2022 and July 2023, participants with newly diagnosed AL according to the WHO classification of hematopoietic tissue (33, 34) were enrolled in the study. Inclusion criteria were participants who had not previously received chemotherapy or radiotherapy, and were suitable for MRI. Exclusion criteria included other vertebral lesions and poor MR images quality. MRI was performed within 1 week after the bone marrow biopsy. The sex, age, and white blood cell (WBC) counts of all AL patients were collected. All enrolled patients were divided into hypo- and normocellular group, moderately hypercellular group, and severely hypercellular group according to bone marrow cellularity (BMC) measured by the BM histology, as described in the “Histological Analysis” section.

### 2.2 MRI acquisition

Conventional DWI and r-FOV DWI of the lumbar spine were performed with a 3.0-T scanner (Discovery 750w, GE Healthcare, Waukesha, WI) and a 32-channel phased-array surface coil in all patients. Each patient took supine position with arms along the body. A bellyband was used for reducing respiratory motions. Conventional DWI and r-FOV DWI sequences were obtained with b-values of 0 and 800 s/mm^2^. The conventional DWI used a 2-dimensional, fat-suppressed, SS-EPI technique with the following parameters: repetition time/echo time, 2000/78.4 ms: section thickness, 4.0 mm; slice spacing,1 mm; FOV, 320 mm × 320 mm; matrix, 128 × 128; number of excitations, 8; bandwidth, 250 kHz; acquisition time, 2min, 32s. The pixel size was 2.5×2.5mm^2^. The r-FOV DWI schemes were based on the description by Saritas et al. (18). A 90° 2-dimensional EPI radiofrequency excitation pulse followed by a 180° refocusing pulse were employed for reducing the FOV in the phase-encoding direction while suppressing the fat signal. The scan parameters were as follows: repetition time/echo time, 2000/78.4 ms: section thickness, 4.0 mm; slice spacing,1 mm; FOV, 320 mm ×128 mm; matrix, 128 × 64; number of excitations, 8; bandwidth, 250 kHz; acquisition time, 1min, 16s. The pixel size was 2.5×2.0mm^2^.

### 2.3 Imaging analysis

All the MRI images were evaluated and processed on the workstation (Advantage Windows 4.6; GE Healthcare). Two radiologists with 10- and 3-years’ experience in musculoskeletal imaging independently performed qualitative and quantitative image quality analyses for r-FOV DWI and conventional DWI, and measured ADC values. Conventional DW, r-FOV DW images, and ADC maps were cross-linked to ensure consistent delineation. Before the evaluation, the conventional DWI images were adjusted by a third radiologist to show the same area as in the r-FOV images for blinded and randomized reading.

#### 2.3.1 Qualitative image quality analysis

Two radiologists qualitatively evaluated the r-FOV DW and conventional DW images using 5-Likert-type scales from 0 to 4 (27):

1. Anatomic structure visualization, sharpness (0 = nondiagnostic,1 = poor, 2 = fair, 3 = good, and 4 = excellent);
2. Distortion was defined as the changes in the vertebral contour (0 = severe distortion, 1 = considerable distortion, 2 = moderate distortion, 3 = slight distortion, 4 = no distortion);
3. Ghosting, motion or susceptibility artifacts (0 = severe artifacts, 1 = considerable artifacts, 2 = moderate artifacts, 3 = slight artifacts, and 4 = no artifacts);
4. overall imaging quality (0 = nonacceptable, 1 = poor, 2 = fair, 3 = good, and 4 = excellent).

#### 2.3.2 Quantitative image quality analysis

In the b = 0 s/mm^2^ midsagittal images of the two DWI sequences, rectangular regions of interest (ROIs) of about 300mm^2^ were placed at the center areas of vertebral cancellous bone from L2 to L4, and ROIs of about 50mm^2^ were placed at the background and the L1/2 disc respectively. The ROIs were copied to b = 800 s/mm^2^ images automatically, and the signal intensity were recorded (26). SNR, contrast-to-noise ratio (CNR) and signal intensity ratio (SIR) were calculated based on the following formulas (22, 35):


$$\text{SNR}=\frac{{\text{S}}_{\text{vertebra}}}{{\text{SD}}_{\text{background}}}\text{ CNR=}\frac{\left|{\text{S}}_{\text{vertebra}}-{\text{S}}_{\text{tissue}}\right|}{\sqrt{{\text{SD}}_{\text{vertebra}}^{2}+{\text{SD}}_{\text{tissue}}^{2}}}\text{ SIR}=\frac{{\text{S}}_{\text{vertebra}}}{{\text{S}}_{\text{tissue}}}$$

where S_vertebra_ is the average signal intensity of the L2 to L4 vertebral bodies, SD_background_ is the standard deviation of background noise, and S_tissue_ stands for the signal intensity of the L1/2 intervertebral disc, SD_vertebra_ and SD_tissue_ represent the standard deviation of vertebral bodies and the L1/2 intervertebral disc, respectively.

#### 2.3.3 ADC values measurement

The ADC quantitative measurement for DWI was performed using the functool software package. Rectangular ROIs of approximately 300 mm^2^ were set in the center areas of vertebral cancellous bone from L2 to L4 in the b = 0 s/mm^2^ midsagittal images. The ROIs were copied to the ADC maps automatically, and the mean value of each ROI was documented. Data in r-FOV DWI and conventional DWI for each participant were expressed as the mean value from L2 to L4.

### 2.4 Histological analysis

Bone marrow biopsy specimens were obtained from the iliac crest. All specimens were fixed in formalin, embedded in paraffin and cut into 4-μm slices for hematoxylin and eosin (H&E) staining. BMC were determined by a pathologist with 20 years of experience using the point counting method as described previously (36, 37). Sixteen randomly chosen fields of the H&E specimen were selected at low magnification and analyzed at a magnification of x400. The selected area is as close as possible to the entire biopsy specimen. A 10×10 square grid was used for counting, and 100 hit targets were documented on each of the fields. Points projected on the hemopoietic cell were scored as one point, and points projected on the lipocyte/hemopoietic cell borders were scored as half a point. The BMC was presented as the mean of the total percentage of hemopoietic cells. Hypocellular, normocellular, moderately hypercellular, and severely hypercellular bone marrow were defined as BMC<35%, 50%>BMC≥35%, 90%>BMC≥50%, and BMC≥90%, respectively (38).

### 2.5 Statistical analysis

Continuous variables are presented as mean ± standard deviation. Interobserver agreements for qualitative image quality scores were evaluated by weighted kappa statistics. Interobserver agreements for SNR, CNR, SIR and ADC values were evaluated using the interclass correlation coefficient (ICC). The image quality scores and quantitative quality parameters between r-FOV DWI and conventional DWI were compared by Wilcoxon signed-rank test. Paired *t*-test was used to compare the ADC values for the two sequences. One-way analysis of variance followed by LSD *post hoc* test was applied for the comparisons of the ADC values among the three groups. Receiver operating characteristic (ROC) analysis was applied for assessing the diagnostic performance of ADC values. DeLong test was utilized to compare the diagnostic performance between the two sequences. Correlation analyses were performed using Spearman correlation. Steiger’s Z test (39) was used to compare the correlations between the two sequences with WBC counts and bone marrow cellularity. Statistical analyses were performed with SPSS statistical software (version 26.0, IBM) and MedCalc statistical software (version 20.0.22). *p*< 0.05 was considered statistically significant.

## 3 Results

### 3.1 Study participants

76 patients with AL underwent r-FOV DWI and conventional DWI in the lumbar spine. 2 patients with vertebral hemangioma, 3 patients with inferior image quality were excluded. 71 patients (40 males and 31 females; mean age ± SD, 44.8 ± 19.1 years; age range, 11–77 years old) were finally enrolled, including 20 patients (9 males and 11 females; mean age ± SD, 49.7 ± 18.6 years; age range, 14–76 years old) with hypo- and normocellular,19 patients (9 males and 10 females; mean age ± SD, 45.0 ± 17.9 years; age range, 11–67 years old) with moderately hypercellular, and 32 patients (22 males and 10 females; mean age ± SD, 41.6 ± 19.9 years; age range, 14–77 years old) with severely hypercellular. There were no significant differences in sex and age among the three groups (*p* =0.160 and *p* =0.337, respectively).

### 3.2 Inter-reader variability

The inter-reader variability results are shown in **Table 1**. All of the qualitative image quality scores and quantitative image quality parameters had good to excellent agreement, and the κ values and ICC values between two radiologists ranged from 0.75 to 0.91. The ICC values of ADC values were 0.92 (95% CI: 0.87–0.95) for r-FOV DWI and 0.85 (95% CI: 0.76–0.92) for conventional DWI, indicating excellent agreement. Therefore, only the first reader’s results were analyzed in our study.

**Table 1:** Inter-reader variability of image quality parameters and ADC value in r-FOV DWI and conventional DWI.

Parameters	r-FOV DWI	Conventional DWI
Anatomical detail	0.84 (0.69–0.92)	0.81 (0.69–0.90)
Distortion	0.87 (0.71–0.90)	0.79 (0.66–0.92)
Artifacts	0.77 (0.62–0.94)	0.75 (0.59–0.89)
Overall imaging quality	0.82 (0.72–0.91)	0.86 (0.72–0.93)
SNR	0.87 (0.79–0.95)	0.82 (0.65–0.91)
CNR	0.80 (0.65–0.92)	0.76 (0.56–0.89)
SIR	0.91 (0.86–0.94)	0.88 (0.70-0.95)
ADC	0.92 (0.87–0.95)	0.85 (0.76–0.92)

Interreader variability is statistically significant (p<0.001).

ADC, apparent diffusion coefficient; CNR, contrast-to-noise; DWI, diffusion-weighted imaging; r-FOV, reduced field-of-view; SIR, signal intensity ratio; SNR, signal-to-noise ratio.

### 3.3 Qualitative and quantitative comparison of image quality

**Figure 1:**
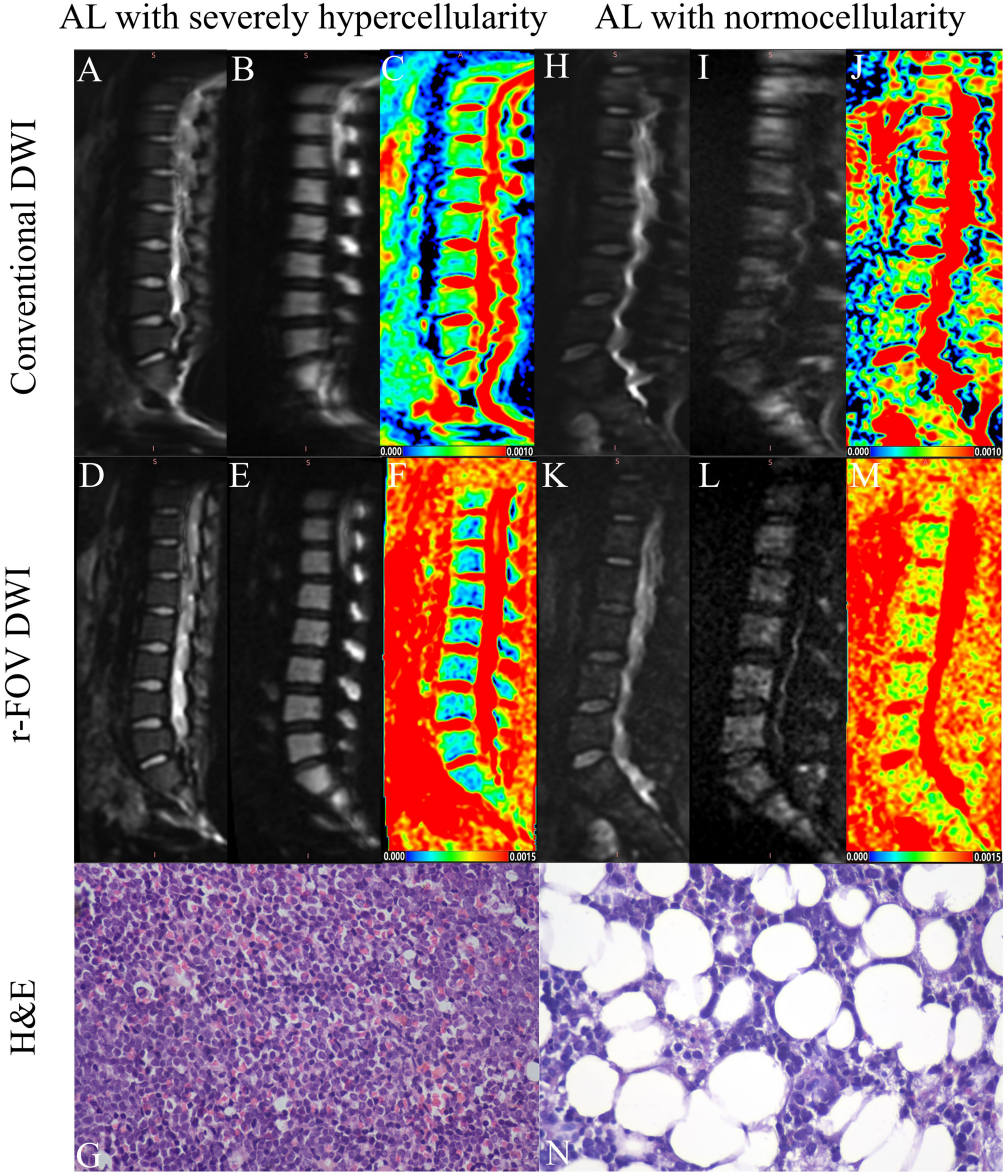
Representative images of an AL patient with severely hypercellularity **(A–G)** and another AL patient with normocellularity **(H–N)**. Compared with the b=0 **(A, H)** and b=800 images **(B, I)** of conventional DWI in the top row, the overall image quality of the conventional DWI **(D, E, K, L)** in the middle row was significantly improved (AL with severely hypercellularity: 2 vs 4; AL with normocellularity: 1 vs 3), exhibiting sharper anatomical structures (AL with severely hypercellularity: 2 vs 4; AL with normocellularity: 1 vs 3), less distortion (AL with severely hypercellularity: 2 vs 3; AL with normocellularity: 1 vs 3) and artifacts (AL with severely hypercellularity: 2 vs 3; AL with normocellularity: 1 vs 3). Parametric maps of conventional DWI **(C)** and r-FOV DWI **(F)** in the AL patient with severely hypercellularity show low ADC values (ADC_c_=0.334×10^-3^mm^2^/sec, ADC_r_=0.397×10^-3^ mm^2^/sec), while parametric maps of conventional DWI **(J)** and r-FOV DWI **(M)** in the AL patient with normocellularity show high ADC values (ADC_c_ = 0.497×10^-3^ mm^2^/sec, ADC_r_ = 0.919×10^-3^ mm^2^/sec). Histological sections of bone marrow were visualized at 400× magnification, **(G)** BMC=95%, **(N)** BMC=47%.

**Figure 2:**
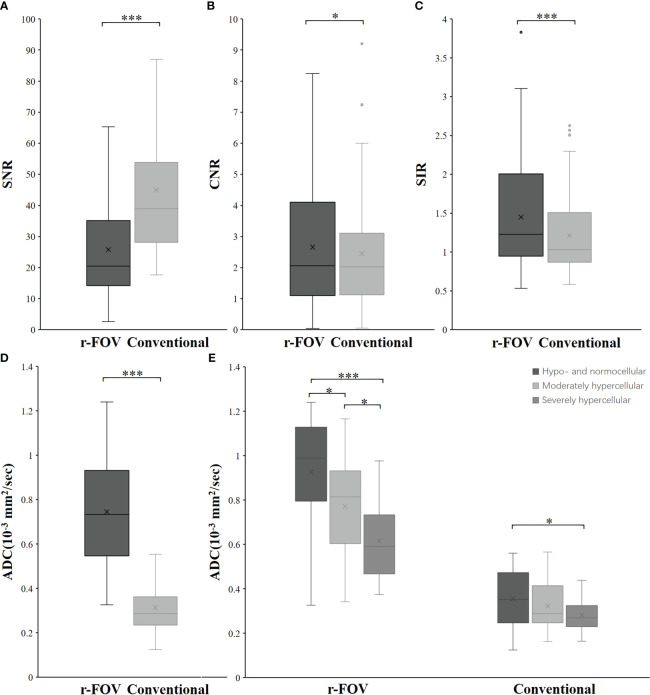
Box-and-whisker plots show the SNR **(A)**, CNR **(B)**, SIR **(C)**, and ADC value **(D)** distributions of r-FOV DWI and conventional DWI. **(E)** Comparisons of ADC values from the two sequences among the hypo- and normocellular, moderately hypercellular, and severely hypercellular groups. *, *p*< 0.05; **, *p*< 0.01; ***, *p*< 0.001.

Representative images of r-FOV DWI and conventional DWI in AL patients are shown in **Figure 1**. The comparisons of qualitative image quality scores and quantitative image quality parameters between conventional and r-FOV DWI are presented in **Table 2** and **Figures 2A–C**. The anatomical detail, distortion, artifacts, and overall imaging quality scores of r-FOV DWI were significantly higher than those of conventional DWI (all *p*< 0.001).

**Table 2:** Qualitative and quantitative comparisons of image quality between r-FOV DWI and conventional DWI in patients with AL.

Image quality parameters	r-FOV DWI	Conventional DWI	*Z*	*p* Value
Anatomical detail	3.08 ± 0.41	1.92 ± 0.37	-7.633	< 0.001
Distortion	3.73 ± 0.45	2.24 ± 0.69	-7.326	< 0.001
Artifacts	3.30 ± 0.49	2.55 ± 0.53	-6.061	< 0.001
Overall imaging quality	3.14 ± 0.39	2.03 ± 0.48	-7.473	< 0.001
SNR	25.81 ± 16.48	45.02 ± 22.07	-7.145	< 0.001
CNR	2.66 ± 1.96	2.46 ± 1.85	-2.114	0.034
SIR	1.42 ± 0.64	1.21 ± 0.50	-6.458	< 0.001

Data are shown as mean ± standard deviation.

AL, acute leukemia; CNR, contrast-to-noise; DWI, diffusion-weighted imaging; r-FOV, reduced field-of-view; SNR, signal-to-noise ratio; SIR, signal intensity ratio.

The CNR and SIR of r-FOV DWI were significantly higher than those of conventional DWI (CNR: 2.66 ± 1.96 vs 2.46 ± 1.85, *Z=*-2.114, *p*=0.034, **Figure 2B**; SIR: 1.42 ± 0.64 vs 1.21 ± 0.50, *Z=*-6.458, *p*< 0.001, **Figure 2C**), while the SNR of r-FOV DWI was significantly lower (25.81 ± 16.48 vs 45.02 ± 22.07, *Z=*-7.145, *p*< 0.001, **Figure 2A**) (**Table 2**).

### 3.4 Quantitative assessment of ADC values for bone marrow infiltration

**Figure 3:**
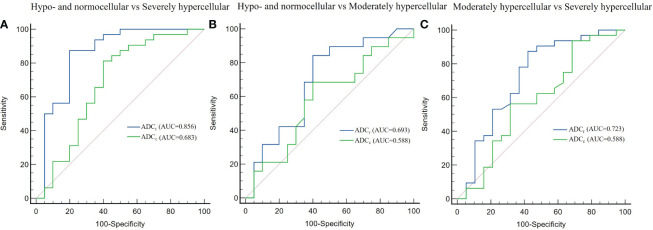
Receiver operating characteristic curves of the diagnostic performance of ADC values in differentiating between hypo- and normocellular group and severely hypercellular group **(A)**, hypo- and normocellular group and moderately hypercellular group **(B)**, moderately hypercellular group and severely hypercellular group **(C)**. ADC_r_ demonstrated significantly better diagnostic performance than ADC_c_ in the differentiation between the hypo- and normocellular group and the severely hypercellular group.

There was significant difference between the ADC value obtained with r-FOV DWI (ADC_r_) and the ADC value obtained with conventional DWI (ADC_c_) (0.74 ± 0.24 ×10^-3^ mm^2^/sec vs 0.31 × ± 0.11 ×10^-3^ mm^2^/sec, *t =*19.914, *p*< 0.001, **Figure 2D**). **Figure 2E** and **Table 3** present the comparison of ADC_r_ and ADC_c_ among hypo- and normocellular, moderately hypercellular, and severely hypercellular groups. ADC_r_ showed statistically significant differences in all pairwise comparisons among the three groups (0.926 ± 0.238 vs 0.772 ± 0.223 vs 0.617 ± 0.164, all *p*<0.05). ADC_c_ in the hypo- and normocellular group was significantly higher than that in the severely hypercellular group (0.356 ± 0.126 vs 0.282 ± 0.077, *p*=0.014). However, ADC_c_ did not show significant differences in the pairwise comparisons of other groups (*p*=0.311 and *p*=0.157, respectively). **Figure 3** depicts the ROC curves of ADC values for evaluating BMC in AL. The corresponding diagnostic characteristics are shown in **Table 4**. In the differentiation between the hypo- and normocellular group and the severely hypercellular group, ADC_r_ demonstrated better diagnostic efficacy than ADC_c_ (Z =2.380, *p* =0.017).

**Table 3:** Comparisons of ADC values from the two sequences among the hypo- and normocellular group, moderately hypercellular group, and severely hypercellular group.

Parameter	Hypo - and Normocellular (n=20)	Moderately hypercellular (n=19)	Severely hypercellular (n=32)	*p Value*	*p^a^*	*p^b^*	*p^c^*
ADC_r_ (10^-3^ mm^2^/sec)	0.926 ± 0.238	0.772 ± 0.223	0.617 ± 0.164	<0.001	<0.001	0.020	0.010
ADC_c_ (10^-3^ mm^2^/sec)	0.356 ± 0.126	0.322 ± 0.114	0.282 ± 0.077	0.036	0.014	0.311	0.157

Data are shown as mean ± standard deviation

a Post hoc paired comparisons between hypo- and normocellular group and severely hypercellular group.

b Post hoc paired comparisons between hypo- and normocellular group and moderately hypercellular group.

c Post hoc paired comparisons between moderately hypercellular group and severely hypercellular group.

AL, acute leukemia; ADC, apparent diffusion coefficient; ADC_c_, apparent diffusion coefficient obtained with conventional diffusion-weighted imaging; ADC_r_, apparent diffusion coefficient obtained with reduced field-of-view diffusion-weighted imaging; CNR, contrast-to-noise; DWI, diffusion-weighted imaging; r-FOV, reduced field-of-view; SIR, signal intensity ratio; SNR, signal-to-noise ratio.

**Table 4:** Diagnostic characteristics of ADC values for evaluating bone marrow cellularity in patients with AL.

Parameters	AUC (95%CI)	Cutoff value	Sensitivity (%)	Specificity (%)	*p* value
Hypo- and Normocellular vs Severely hypercellular					
ADC_c_	0.683(0.539, 0.805)	0.328	81.25	60.00	0.030
ADC_r_	0.856(0.731, 0.938)	0.790	87.50	80.00	<0.001
Hypo- and Normocellular vs Moderately hypercellular					
ADC_c_	0.588(0.419,0.743)	0.318	68.42	60.00	0.350
ADC_r_	0.693(0.525, 0.831)	0.952	84.21	60.00	0.027
Moderately hypercellular vs Severely hypercellular					
ADC_c_	0.588(0.441,0.724)	0.380	93.75	31.58	0.321
ADC_r_	0.723(0.580, 0.839)	0.790	87.50	57.89	0.006

AL, acute leukemia; ADC, apparent diffusion coefficient; ADC_c_, apparent diffusion coefficient obtained with conventional diffusion-weighted imaging; ADC_r_, apparent diffusion coefficient obtained with reduced field-of-view diffusion-weighted imaging; AUC, area under the curve; CI, confidence intervals.

Correlations between ADC values and BMC as well as WBC counts are presented in **Table 5**. The ADC values of both r-FOV DWI and conventional DWI showed negative correlations with BMC (r-FOV: *r*=-0.546, *p*<0.001; Conventional: *r*=-0.262, *p*=0.027, respectively) and WBC counts (r-FOV: *r*=-0.617, *p*<0.001; Conventional: *r*=-0.357, *p* = 0.002, respectively). Correlations between ADC_r_ with BMC and WBC counts were significantly higher than those between ADC_c_ with BMC (Z=-2.008, *p* = 0.045) and WBC counts (Z=-2.022, *p* = 0.043).

**Table 5:** Comparisons of the correlations between ADC values obtained with the two sequences and BMC, and WBC counts.

Indicators	ADC_c_		ADC_r_		Z	*p*
	*r* (95%CI)	*p*	*r* (95%CI)	*p*		
BMC	-0.262(-0.485, -0.026)	0.027	-0.546(-0.717, -0.347)	< 0.001	-2.008	0.045
WBC	-0.357(-0.569, -0.116)	0.002	-0.617(-0.744, -0.483)	< 0.001	-2.022	0.043

ADC_c_, apparent diffusion coefficient obtained with conventional diffusion-weighted imaging; ADC_r_, apparent diffusion coefficient obtained with reduced field-of-view diffusion-weighted imaging; BMC, bone marrow cellularity; CI, confidence intervals; WBC, white blood cell.

## 4 Discussion

DWI is a non-invasive imaging technique that could reflect tissue cellularity (2, 6, 7), and it has been applied to differential diagnosis, response evaluation, and prognosis prediction in malignant bone marrow diseases (3–5). Our study demonstrated r-FOV DWI had significantly preferable subjective image quality and higher CNR, SIR compared with conventional DWI, despite lower SNR. In addition, ADC_r_ had better performance in assessing BMC and a higher correlation with BMC as well as WBC than ADC_c_. These findings suggest that r-FOV DWI is a promising technique for reducing image artifacts, improving image quality, and assessing bone marrow infiltration in AL.

MRI is more frequently applied than other imaging modalities in assessing the bone marrow microenvironment of hematological malignancies. MR spectroscopy (40, 41), chemical-shift imaging (42), intravoxel incoherent motion (IVIM) diffusion-weighted MRI (32, 43), arterial spin labeling (44), and dynamic contrast-enhanced MRI (45) have been used to quantify water-fat composition, cellularity, and blood perfusion changes in bone marrow, respectively. Compared with these techniques, DWI has the advantages of simple imaging technique, short scanning time, and no need for injection of contrast agent, which is more suitable for clinical practice. Nevertheless, conventional DWI based on SS-EPI technique is susceptible to field inhomogeneities, local gradients, and chemical shift effects. r-FOV DWI has been utilized to various organs such as gallbladder, uterus, prostate, pancreas, nasopharynx, breast, and rectum (21–31). Some researchers suggested that the spine is well suited to r-FOV DWI, because of its elongated anatomical structure, heterogeneous bone density, and air cavities in the abdomen, and their studies have shown the availability of r-FOV DWI for the spine (18–20). Despite that, the authors did not objectively and quantitatively evaluate the r-FOV DW image quality, nor did they compare the performance in the evaluation of bone marrow diseases between r-FOV and conventional DWI. We compared the image quality between the two sequences from both qualitative and quantitative aspects and demonstrated that r-FOV DWI was superior in assessing bone marrow infiltration of AL.

Our results suggested the overall image quality was improved on r-FOV DWI with sharper anatomical structure, reduced distortions and artifacts, and higher CNR as well as SIR when compared with conventional DWI. Similar findings were found in previous studies where the r-FOV DWI technique was used to the gallbladder carcinoma (21), cervical carcinoma (22, 23), nasopharyngeal carcinoma (26), pancreas lesions (27) and rectal carcinoma (29). By reducing the FOV in the phase-encoding direction, r-FOV DWI has a faster traverse of k-space and a higher bandwidth, which enables it to achieve a higher spatial resolution in the same scan time and decrease image artifacts such as blurring and pixel misregistration (18–20). Distortions can be reduced by decreasing the number of acquisition steps and shortening the length of the EPI echo train on r-FOV DWI with a spatially selective RF pulse (18). However, our quantitative results showed r-FOV DWI is disadvantaged in terms of SNR. SNR is commonly inversely proportional to the FOV size. The SNR of the r-FOV would degrade unless the acquisition time was prolonged, as observed in gallbladder carcinoma (21), cervical carcinoma (22), and endometrial cancer (24). It has also been found in some studies that the SNR of r-FOV is significantly higher or comparable to conventional sequences (23, 25, 26), mainly because the SNR is a relative measurement that is determined by various factors, such as FOV, matrix, echo time, signal average, b-values, magnetic field strength, and T_2_ value for the tissue (23, 35). Consequently, it is crucial to consider the balance between SNR and spatial resolution as well as acquisition time when setting the scanning parameters to achieve the satisfactory imaging quality.

In our study, the ADC values from r-FOV DWI were higher than those from conventional DWI, which is accordant with some prior studies (24–26). r-FOV DWI has better spatial resolution, desired fat suppression effects, and less artifacts, so its ADC values may be more accurate. However, some studies reported that the ADC values of r-FOV DWI did not differ statistically (19–22, 29), while others reported significantly lower values than conventional DWI (23, 28, 30). The discrepancy could be explained by the differences in magnetic field strength, imaging technology vendor, b values, echo time, and organs under analysis in the various studies (26, 28).

Hypercellularity by leukemia cells decreased the extracellular space and water proton mobility, causing enhanced signal intensity on DW images and reduced ADC value. In our study, ADC_r_ can distinguish three groups of cellularity with different degrees, while ADC_c_ was only able to distinguish the hypo- and normocellular group and the severely hypercellularity group. Furthermore, ADC_r_ demonstrated better diagnostic efficacy than ADC_c_ in differentiating between the hypo- and normocellular group and the severely hypercellular group. WBC is a clinical marker of tumor burden in AL. Although ADC values from both conventional DWI and r-FOV DWI were negatively correlated with BMC and WBC counts, ADC_r_ showed significantly higher correlations than ADC_c_. Accordingly, r-FOV DWI could be a superior alternative to conventional DWI in evaluating bone marrow infiltration of AL.

This study had several limitations. First, this was a single-center study with a relatively small sample size. Second, we did not acquire or compare axial images of the lumbar spine, which may show more anatomical structures used for image quality evaluation and better visualize extramedullary infiltration. However, sagittal images most adequately displayed the vertebral body area of interest with the fewest images, and they are most commonly used for the assessment of lumbar bone marrow. Third, the biopsy samples were obtained from iliac marrow, which were inconsistent with the ROIs of MRI. AL was a systemic disease involving the whole-body marrow including the ilium and lumbar. Thus, there may be similar histological characteristics in the patients’ ilium and lumbar vertebrae. Finally, ADC derived from DWI cannot separate diffusion and microcapillary blood flow information. Reduced FOV IVIM DWI would be used to remove the effect of tissue microcapillary perfusion in the next study.

In conclusion, compared with conventional DWI, r-FOV DWI provides significantly improved image quality of the lumbar spine in AL patients, thus yielding preferable performance in assessing bone marrow infiltration.

## Data availability statement

The raw data supporting the conclusions of this article will be made available by the authors, without undue reservation.

## Ethics statement

The studies involving humans were approved by Ethics Committee of the Second Hospital of Shanxi Medical University. The studies were conducted in accordance with the local legislation and institutional requirements. The participants provided their written informed consent to participate in this study.

## Author contributions

WB: Conceptualization, Writing – original draft, Writing – review & editing, Investigation. LW: Data curation, Methodology, Writing – original draft. JL: Data curation, Methodology, Writing – original draft. SC: Data curation, Methodology, Writing – original draft. WW: Formal analysis, Methodology, Writing – original draft. RF: Formal analysis, Methodology, Writing – original draft. JN: Conceptualization, Project administration, Supervision, Writing – review & editing.
